# Live Poultry Market Closure and Control of Avian Influenza A(H7N9), Shanghai, China

**DOI:** 10.3201/eid2009.131243

**Published:** 2014-09

**Authors:** Yi He, Peihong Liu, Songzhe Tang, Yong Chen, Enle Pei, Baihui Zhao, Hong Ren, Jian Li, Yiyi Zhu, Hongjin Zhao, Qichao Pan, Baoke Gu, Zhengan Yuan, Fan Wu

**Affiliations:** Shanghai Municipal Center for Disease Control and Prevention, Shanghai, China (Y. He, S. Tang, Y. Chen, B. Zhao, H. Ren, J. Li, Y. Zhu, Q. Pan, B. Gu, Z. Yuan, F. Wu);; Shanghai Municipal Animal Disease Control Center, Shanghai (P. Liu, H. Zhao);; Shanghai Wildlife Conservation and Management Center, Shanghai (E. Pei)

**Keywords:** avian influenza, influenza, China, intervention, H7N9, live poultry markets, closure, respiratory infections, LPMs, viruses, influenza viruses

**To the Editor:** China reported its first human infections with avian influenza A(H7N9) virus in late March 2013 (*1*). In the following weeks, 131 human infections were confirmed; 33 occurred in Shanghai (http://www.moh.gov.cn). Because infection with this novel virus had a high fatality rate and posed a pandemic risk, Shanghai disease control authorities launched rapid investigations to identify the source of the infections. Migratory birds, mammals, poultry, and humans could be potential reservoirs of H7 subtype avian influenza viruses (*2*,*3*), so all of these possibilities were simultaneously evaluated immediately after the discovery of the novel virus.

To investigate human-to-human transmission, we evaluated 45 close contacts of the first 6 reported case-patients. The only suspected human-to-human transmission was in 1 family cluster with 2 confirmed cases. Intensive follow-up monitoring (20 contacts of this cluster and 25 of the other 4 case-patients) did not identify any further infections, which suggests that sustained human-to-human transmission did not occur.

Migratory birds are natural reservoirs for avian influenza viruses (*2*,*4*), and Shanghai is in the eastern Asia–Australian migratory shorebird flyway. Thus, transmission of these viruses from wild birds to humans is possible. The Shanghai forestry authority has conducted surveillance for influenza virus among migratory birds since 2004. During January 2010–April 2013, a total of 884 throat/cloacal swab, serum, and fecal samples from 496 birds were tested, and no infections with influenza viruses of subtypes H7 or N9 were found. After human infections with influenza A(H7N9) virus occurred, an additional 229 samples were collected from migratory birds at Chongming Island, Shanghai Zoo, and Shanghai Wildlife Park. All were negative for the virus.

Avian influenza surveillance in domesticated animals (e.g., minks, raccoons, tigers, and pigs) in high-risk regions of Shanghai has been conducted since 1995. During 2010–2012, a total of 13,691 samples from these animals were tested, and all were negative for H7 subtype influenza viruses. After human infections with influenza A(H7N9) virus were identified, another 1,129 samples were collected from domesticated mammals for enhanced surveillance; all were negative for H7 subtype influenza viruses.

Live poultry was also considered a potential source of influenza A(H7N9) infection because poultry in wet markets have been previously found to be infected with H7 subtype avian influenza viruses (*5*–*7*). Shanghai residents could be exposed to influenza A(H7N9) virus from live poultry in 2 ways: by raising poultry at home or by visiting live poultry markets (LPMs). To assess the risk from home-raised poultry, we collected 405 blood, cloacal/throat swab, and environmental samples from poultry raised in family courtyards or in communities surrounding the residences of the first 6 identified case-patients. The samples were tested for antibodies to H7 subtype viruses and RNA for subtype H7N9 virus, and all results were negative.

Previous studies have shown that LPMs are potential locations for virus transmission through human-poultry contact (*8*,*9*). Our field investigation of the 6 initially confirmed influenza A(H7N9) case-patients revealed that 2 had visited LPMs, 2 had direct contact with live poultry from LPMs, and 1 had experienced both exposures. To assess the risk for infection at LPMs, we collected 280 water, soil, cloacal swab, and throat swab samples from the LPMs surrounding the locations of the initial 6 cases. PCR testing found that 20 samples were positive for influenza A(H7N9) virus. These results indicate that the human case-patients were most likely infected from poultry in LPMs.

On the basis of the epidemiologic and laboratory evidence, the Shanghai municipal government temporarily closed all 464 LPMs on April 6, 2013. The markets were cleaned and disinfected, and relevant authorities in the neighboring provinces were notified of the action and requested not to transport live poultry to Shanghai. The municipal government reimbursed the poultry traders and raisers for these measures.

After the closure of LPMs, 4 additional human influenza A(H7N9) cases were detected in Shanghai within the first 7-day incubation period, and no new cases were detected during the rest of 2013, although other influenza virus subtypes continued to be detected. Meanwhile, in other provinces where closure of LPMs was not implemented in a timely manner, 31 cases were reported during the first incubation period and 13 during the second incubation period (Figure). In addition, Shi et al. reported that isolates from the LPMs showed 99.1%–99.9% nucleotide sequence homology with isolates from 2 of the first 6 human case-patients (*10*), which further indicates that the virus spread from infected poultry in the LPMs.

**Figure:**
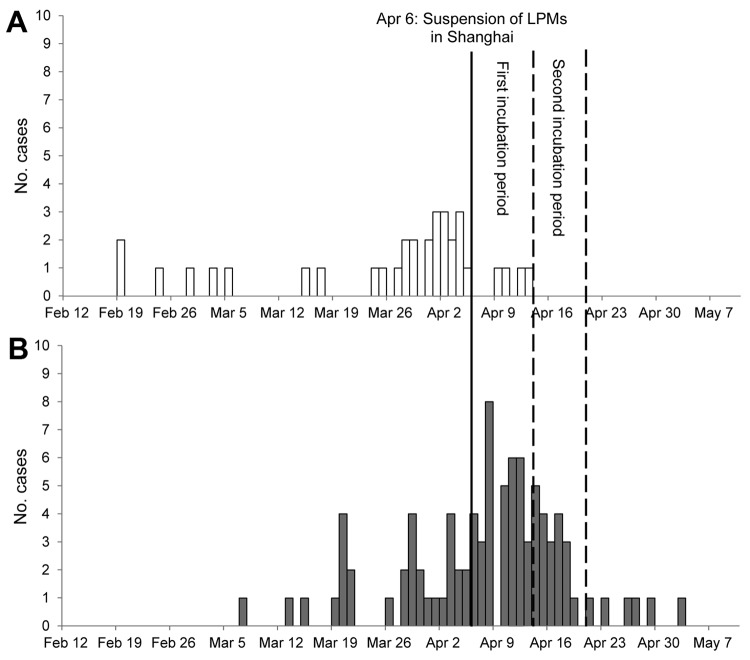
Illness onset dates for 33 confirmed cases of influenza A(H7N9) infection in Shanghai (A) and 78 cases in other provinces (B) in China, February 12–May 10, 2013. Solid vertical line indicates date live poultry markets (LPMs) in Shanghai were suspended (April 6, 2013). Dashed vertical lines delineate first and second incubation periods.

The closure of LPMs after epidemiology and laboratory investigations proved timely and effective in the control of human infection with this novel virus. In early 2014, Shanghai lifted the ban on LPMs. Eight new influenza A(H7N9) cases were identified in early January 2014, but LPMs were again closed on January 31, 2014, and no further cases have been reported. The long-term effectiveness of the LPM closures remains to be determined.
